# Factors associated with clinician adherence to guidelines for postpartum care: results from a California survey

**DOI:** 10.1186/s12884-025-07362-0

**Published:** 2025-03-13

**Authors:** Sylvia Guendelman, Serena Xinzi Wang, Maureen Lahiff, Hayley E. Miller, Lawrence Lurvey

**Affiliations:** 1 School of Public Health, University of California, Berkeley, 2121 Berkeley Way, Room 6124, Berkeley, Ca 94720-7360 USA; 2 School of Public Health, University of California, Berkeley Class of 2025, 2121 Berkeley Way West, Room 5302, Berkeley, Ca 94720-7360 USA; 3 School of Public Health, University of California, Berkeley, 2121 Berkeley Way, Room 5302, Berkeley, Ca 94720-7360 USA; 4 Department of Obstetrics, Gynecology & Reproductive Sciences, Division of Maternal Fetal Medicine, University of California, San Francisco, 490 Illinois St. 10th floor, San Francisco, Ca 94158 USA; 5 Kaiser Permanente Los Angeles Medical Center, 4867 Sunset Blvd, Los Angeles, Ca 90027 USA

**Keywords:** Postpartum care, Adherence to clinical practice guidelines, ACOG recommendations, Medical, social and behavioral care practices, Obstetric care, Facilitators and barriers to adherence

## Abstract

**Background:**

In 2018, the American College of Obstetricians and Gynecologists (ACOG) issued multiple recommendations to optimize care within the first 12 weeks postpartum. We explored the extent to which clinicians follow ACOG’s recommendations at the first postpartum visit and identified factors associated with overall adherence to clinical recommendations.

**Methods:**

Between September 2023 and February 2024, we collected data from an online survey about the care practices of 174 obstetric clinicians practicing in California. The response rate was 76%. Adherence was measured by asking the extent to which clinicians always complete care components drawn from a list of 26 of ACOG’s clinical recommendations. We used descriptive statistics and ran linear regression models to quantify the association between adherence to guidelines and years of clinical practice, proportion of patients utilizing Medi-Cal, the method of reimbursement received by the provider, perceived organizational, financial and patient barriers, duration of the visit and number of collaborations with other providers.

**Results:**

The median percentage of components checked by clinicians was 62%. Significantly higher adherence was found among clinicians with at least 12 years of practice compared with those with approximately 5 years or less and among clinicians who collaborated with 5 or more multidisciplinary providers versus fewer than 3. Adherence was also higher among clinicians who on average spent at least 30 min vs. less than 20 min with their patients and those who perceived high financial barriers to care among their patients. In contrast, clinicians who served highly mixed practices of Medi-Cal and non-Medi-Cal recipients reported lower adherence.

**Conclusion:**

We sought to understand which clinicians were more able to align their practice with ACOG guidelines at the first postpartum visit. We found that more experienced clinicians, those who simplified their practices to either all Medi-Cal or few Medi-Cal recipients, and those who collaborated more with other providers from various disciplines were best able to provide the care recommended in the guidelines. Our findings highlight where policy, resources and training are needed to improve guideline adherence and whole person care.

**Supplementary Information:**

The online version contains supplementary material available at 10.1186/s12884-025-07362-0.

## Background

In 2018, the American College of Obstetricians and Gynecologists (ACOG) issued multiple recommendations to optimize care in the first 12 weeks postpartum. The recommendations recognized the importance of the postpartum period for the health and wellbeing of mothers, other birthing people and their infants [1–3]. 

ACOG practice guidelines include a broad set of recommendations spanning clinical care elements, family planning, infant health, social and behavioral components, and planning for future preventative care [2–3]. Evidence for clinician compliance with these recommendations is both limited and timely, given increasing rates of maternal mortality and morbidity, large racial inequities in perinatal outcomes, and growing behavioral health problems [2–3]. Data from the 2020 Maternal Mortality Review Committees indicate that 63% of pregnancy-related deaths occurred in the postpartum period and the most frequent underlying causes of pregnancy-related deaths were mental health conditions, cardiovascular conditions, infection, hemorrhage, embolism, and hypertensive disorders [4]. Using a bundle approach that combines sets of evidence-based practices to improve health outcomes, and perinatal quality collaboratives, where hospitals teach each other the bundles, has been shown to be effective in reducing perinatal morbidity and mortality [5]. ACOG thus designed their postpartum guidelines to bundle together initiatives that also take a holistic, patient-centered approach. It follows that compliance with the entirety of the ACOG guidelines should be the most effective way to improve maternal outcomes and avoid postpartum morbidity and mortality.

In a recent study of obstetric clinician care priorities and care practices at the first postpartum visit, we found that clinicians rarely perform all the postpartum care components recommended by ACOG [6]. Our survey findings demonstrated that clinicians face barriers completing the recommendations, especially those components related to social drivers of health. However, clinicians are more likely to perform components that they prioritize highly—namely depression and anxiety, breast health/breastfeeding issues, vaginal birth complications and family planning counseling [6]. 

What barriers clinicians face in implementing multiple ACOG recommendations remains largely unexplored. A study of obstetricians in Louisiana identified systemic barriers (e.g. overbooked schedules and unclear roles of providers for postpartum visits) and patient-related barriers (e.g. patient no-shows, transportation and childcare challenges) as important perceived barriers to implementing the recommendations [7]. 

Another small qualitative study of diverse providers in Chicago revealed multiple systemic barriers such as insurance barriers to specialists and primary care providers, insufficient resource coordination support, provider discontinuity, scheduling challenges and a large nonclinical task burden [8]. Low health literacy, lack of trusting relationships with providers and language barriers were noted patient-related barriers [8]. Neither study used statistical models to adjust for covariates.

ACOG claims that reimbursement policies that do not support postpartum care as an ongoing process of care may act as another barrier to comprehensive delivery of care [1]. Evidence suggests that the implementation of clinical guidelines is associated with increased provider satisfaction with the care they provide [9–10]. Nonetheless, too many professional society recommendations in the face of limited time and resources could result in a mismatch between expectations and reality, leading to clinician dissatisfaction with the quality of care provided [11]. While ACOG provides general guidelines to make care more patient-centered and comprehensive, it does not provide guidance on how to prioritize specific components in limited postpartum visits. Hence, the extent to which clinicians are adhering to these guidelines to enhance their performance and quality of care warrants examination.

The aim of this exploratory study is to identify factors that are associated with the degree of adherence to ACOG recommendations among obstetric care clinicians in California. Using a contemporary cohort of practicing clinicians (mainly obstetrician gynecologists, midwives and family medicine doctors) we explore the following research questions:

1. To what extent do clinicians “always adhere” to the practice guidelines at the first postpartum visit?
2. Is the degree of adherence to the ACOG recommendations associated with clinicians’ years of practice and other practice environment characteristics, the time spent with the patient, the perceived systemic, financial and patient-related barriers to care, their collaborative work efforts and/or the type of reimbursement that clinicians receive for their services?
3. Do the associations remain when we adjust for other covariates in regression models?
4. Are the factors associated with adherence to care of patients’ medical needs similar to those factors associated with adherence to care of behavioral and social needs of patients?

A better understanding of the factors that incentivize (or impede) overall adherence and adherence to specific sets of components related to medical care and care of behavioral and social needs can help us identify effective interventions to boost adherence and improve performance, standardize patient care and improve maternal-infant health outcomes in the postpartum period.

## Materials and methods

To address the research questions, we followed a conceptual model based on the Cabana framework for evaluating adherence to clinical guidelines [12]. This framework acknowledges that multiple barriers can limit adherence, some of which constitute internal barriers and others are external barriers. Internal barriers relate to individual providers such as lack of awareness, knowledge and skills or agreement with the guidelines; external barriers are associated with barriers from patients, the practice environment, payers, or other forces that are beyond the clinicians’ control [12]. 

**Fig. 1:**
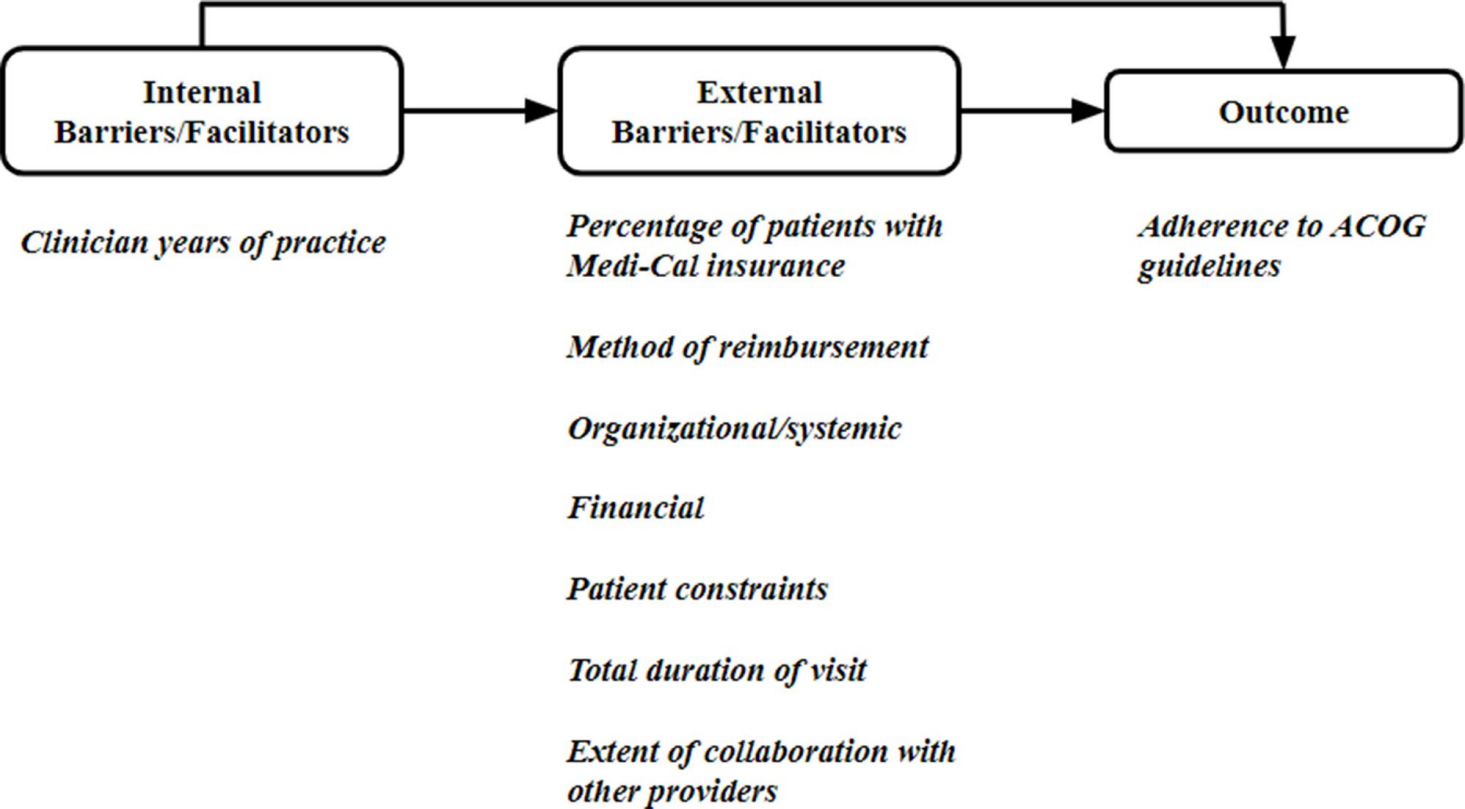
Potential contributors to guideline adherence

Our conceptual model includes one internal barrier, namely years of practice, as an indicator of clinicians’ confidence and skills built over time. Evidence suggests that lack of adherence to guidelines is linked to a lack of knowledge and awareness of practice guidelines, which are more common among trainees with fewer years of practice than among practicing physicians [13]. The other barriers are external. They include the method of provider reimbursement as an indicator of whether it supports postpartum care; proportion of patients covered by Medi-Cal insurance (California’s Medicaid program) as an indicator of the additional medical, behavioral care and social needs of disadvantaged populations; perceived organizational/systemic, financial and patient-related barriers; the amount of time spent with the patient at the first postpartum visit as an indicator of time resources available to the clinician and the extent of collaboration with other providers as a marker of an integrated care practice (Fig. 1). The conceptual model recognizes that while several barriers can hinder the uptake of ACOG recommendations, the absence of these barriers can facilitate uptake. Furthermore, it focuses on aggregate measures of adherence to care components and does not drill down to specific components.

### Study design

This cross-sectional study used data from the California Postpartum Care Survey, an anonymous, 32-question online survey of current obstetric-care practices in the postpartum, perceived care priorities, and barriers and opportunities to improve care. Details about the survey questions, recruitment and informed consent have been reported in our previous paper [6]. The data were collected using Qualtrics software between September 1, 2023, and February 2, 2024. To be eligible for the survey, participants had to be active clinicians providing obstetric care, including postpartum care, in California. Of the 353 potential respondents who opened the link to the survey, 124 were excluded from analysis because they did not meet the eligibility criteria, or their eligibility was unknown. An additional 55 eligible respondents were excluded because they did not complete at least 95% of the survey questions, yielding a sample of 174 participants. Based on the eligible respondents, the overall response rate was 76%. Survey respondents did not differ from eligible non-respondents by clinician type. Clinicians included OB/GYNs (*n* = 97), midwives (*n* = 61), family medicine doctors (*n* = 12), and nurse practitioners (*n* = 4). Compared to the population of active OB/GYNs in California in 2015–2020, our OB/GYN survey respondents were more likely to be younger than age 35 (19.6% vs. 5%), female (91% vs. 64%) and white non-Hispanic (62.9% vs. 38%), although the proportion of Black (9.3% vs. 5.6%) and Hispanics (6.2% vs. 7.9%) were quite comparable [14]. Midwives in our survey were more likely to be over age 65 than the population of practicing midwives in California (14.8% vs. 8.2% among nurse midwives and 11.1% among licensed midwives) [15, 16]. Consistent with California’s midwifery population, midwives in our survey were predominantly white (77% vs. 76%) and female (96.7% vs. 97.2%) [15, 16]. The study was approved by the Institutional Review Boards of the University of California, Berkeley (Protocol No.2023-01-15992) and Stanford University (Protocol No. 71687).

### Survey measures

Overall adherence to ACOG guidelines at the first postpartum visit was the outcome. It refers to the proportion of components that were “always completed” out of a total checklist of 26 care components drawn from ACOG postpartum care practice recommendations [1–3]. Adherence was defined by the response “yes, always checked” from the survey question “*Do you check for the following elements at the first postpartum visit?”* To measure adherence to specific sub-groups, the 26 care components were categorized into two broad groups: medical needs which refer to the biological/genetic explanations of physical conditions and the clinical interventions to avoid or reduce risk of deterioration and recurrence; and behavioral care/social needs that arise from the conditions in the environment in which birthing people are born, interact, work and live that affect their health [17]. 

As shown in Table 1, the 11 care components indicating medical needs were: *c-section birth complications*,* vaginal birth complications*,* pregnancy-related complications*,* physical recovery after labor*,* chronic health conditions*,* family planning counseling*,* contraceptive provision*,* resume sexual activity*,* review birth experience and prepare for future pregnancies*,* development/implementation of a postpartum care plan and transitioning to primary care based on obstetric history that details patient’s needs.* The 15 behavioral care and social components were: *depression and anxiety*,* substance use*,* smoking*,* maternal sleep*,* exercise and nutrition*,* diet and weight trajectory*,* social and emotional support*,* intimate partner violence*,* safe work environment*,* work evaluation*,* adverse childhood experiences (ACEs evaluation)*,* food and housing insecurity*,* breast health/breastfeeding*,* infant safe sleep and infant bonding.* Since our survey focused on maternal health, the components related to breast health/infant feeding, infant safe sleep and infant bonding are considered socio-emotional rather than medical.

**Table 1:** Medical and behavioral/social needs care components

Medical care components	Behavioral/social needs care components
• C-section birth complications • Vaginal birth complications • Pregnancy-related complications • Physical recovery after labor • Chronic health conditions • Family planning counseling • Contraceptive provision • Resuming sexual activity • Review birth experience and prepare for future pregnancies • Development/implementation of a postpartum care plan • Transitioning to primary care	• Depression and anxiety • Substance use • Smoking • Maternal sleep • Exercise and nutrition • Diet and weight trajectory • Social and emotional support • Intimate partner violence • Safe work environment • Evaluate work environment • ACEs evaluation • Food and/or housing insecurity • Breast health, breastfeeding issues • Infant safe sleep • Infant bonding

Following our conceptual model, the main independent variables were: the clinician years of practice, grouped into four quartiles and coded using indicator variables (< 5.25, ≥ 5.25-<12, ≥ 12-<22, ≥ 22); the proportion of patients with Medi-Cal insurance served, grouped into four categories (< 25%, 25–50%, 51–75%, > 75%); the method of reimbursement received by the clinician categorized as fee-for service, global payment or fixed salary; and several types of perceived barriers obtained by asking the clinician “How often do you encounter [each of] the following barriers when providing postpartum care: *Organizational/systemic*: e.g. poor care coordination, limited access to referrals, limited clinic hours, excessive paperwork; *Financial*: e.g. insufficient or slow reimbursement, insurance disruptions; *Patient constraints*: e.g. limited time, lack of knowledge, language differences, distrust.” Responses to each of these barriers were first measured with a 5-point Likert scale ranging from never to always and were re-categorized as infrequent (always or seldom), sometimes, and frequent (often or always). Other barriers/facilitators were the average duration in minutes of the first postpartum visit, grouped into tertiles (< 20, ≥ 20-<30, ≥ 30) and the number of providers that the clinician collaborates with. The latter was obtained by asking “Who do you currently collaborate (i.e. work regularly with, send/receive referrals and/or coordinate patient care) with during the postpartum period of care?” The list of collaborators included doulas, lactation consultants, nutritionists, psychiatrists, psychologists or therapists, social workers, and “others.”

Other variables of interest were type of clinician (obstetrician/gynecologists, midwives, or family practitioners), type of practice setting, and location of practice.

### Data analysis

**Fig. 2:**
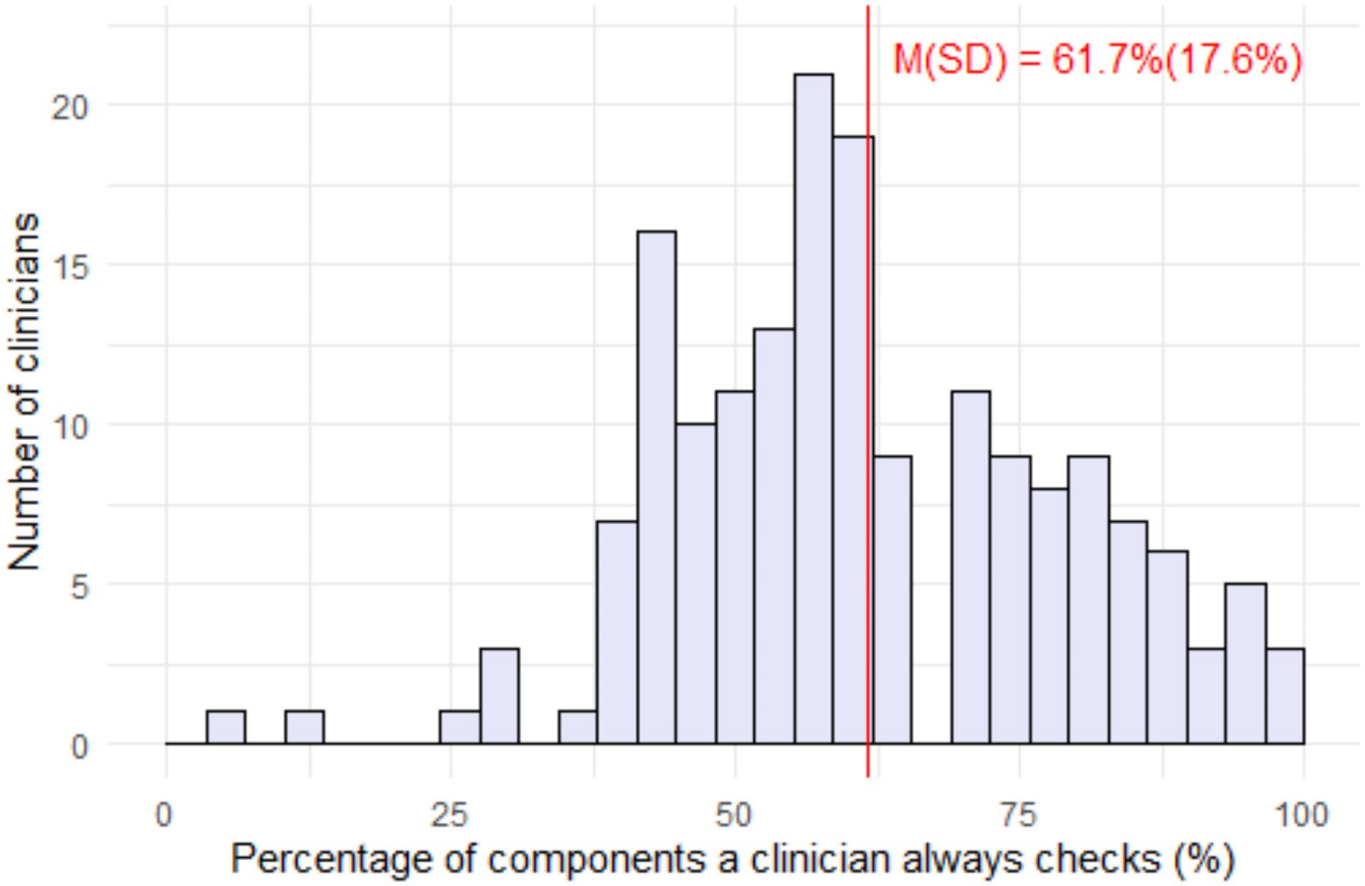
Proportion of care components that clinicians always check

To examine the extent to which clinicians always adhere to the guidelines we calculated the mean, median and range of the percent of care components that were adhered to and display its distribution in a histogram (Fig. 2). We then performed bivariate analyses to test the association between the independent variables and the extent of adherence using overall F-tests for association (Table 2). P-values less than 0.05 were considered of statistical significance.

**Table 2:** Characteristics associated with adherence to ACOG guidelines among obstetric care clinicians in California: bivariate analyses

Characteristics	*n* ^†^	Percentage of components clinicians always check (%) Mean(SD)	*p*-value*
**All**	174	61.71(17.62)	
**Clinician type**			N.S.
OB/GYNs	97	59.12(17.57)	
Midwives	61	63.92(17.27)	
Family medicine doctors	12	67.95(17.83)	
Nurse practitioners	4	72.12(16.75)	
**Practice setting**			N.S.
University/Academic	50	59(16.13)	
Community hospital/health center/clinic	41	67.36(20.31)	
Staff model HMO	25	56.76(17.56)	
Private practice	39	61.34(16.34)	
Multi-affiliated/Other	18	62.6(15.14)	
**Practice location**			N.S.
Urban, large city	87	61.94(17.25)	
Suburb, near large city	58	58.69(19.67)	
Small city or town	28	67.57(12.93)	
**Years of practice**			< 0.01
< 5.25	43	57.07(14.92)	
≥ 5.25 to < 12	40	56.54(18.81)	
≥ 12 to < 22	42	64.28(16.72)	
≥ 22	45	67.27(17.85)	
**Percentage of Medi-Cal insured patients**			< 0.05
< 25%	61	63.18(17.62)	
25–50%	37	60.71(16.39)	
51–75%	28	54.81(15.69)	
> 75%	41	66.98(19.11)	
**Method of clinician reimbursement**			< 0.05
Fee for service	25	66.15(22.21)	
Global maternity fee	91	63.78(16.77)	
Fixed salary	53	56.96(15.9)	
**Organizational barriers to care**			N.S.
Infrequent	32	63.34(16.81)	
Sometimes	47	64.16(15.37)	
Frequent	93	60.13(18.97)	
**Financial barriers to care**			< 0.05
Infrequent	57	59.05(16.92)	
Sometimes	56	58.86(18.53)	
Frequent	59	67.33(16.45)	
**Patient barriers to care**			N.S.
Infrequent	41	61.91(13.91)	
Sometimes	69	62.1(19.65)	
Frequent	62	61.47(17.79)	
**Duration of visit (min)**			< 0.05
< 20	40	57.98(20.31)	
≥ 20 to < 30	65	59.06(17.96)	
≥ 30	69	66.38(14.59)	
**Extent of collaboration with other providers**			< 0.01
≤ 2	33	57.46(12.56)	
3	39	60.94(16.64)	
4	41	55.53(14.56)	
≥ 5	61	68.67(20.22)	

*N.S. indicates a non-significant result (p-value > 0.05) from overall F-tests

^†^The total n varies for some variables due to missing values

The variables that were significantly associated with the outcome were selected as covariates for regression models. We ran simultaneous linear regression models to quantify the association between all the independent variables and the outcome, while adjusting for the other covariates in the model (Table 3, full model). To avoid overfitting the model, we also ran restricted models dropping the independent variables that did not significantly contribute to the outcome, one at a time. We present the model that shows the best fit according to the adjusted R^2^ (Table 3, model 2). Finally, we performed similar regressions to quantify the association between the independent variables and (1) adherence to components indicating medical needs and (2) adherence to behavioral care and social components (Table 4). We present regression coefficients that show the mean change in the outcome given a unit shift in the independent variable, and p-values from the full and restricted models.

**Table 3:** Characteristics associated with adherence to ACOG guidelines: regression coefficients and p-values from linear regression models

	Model 1 *		Model 2 **	
Sample characteristics	Coefficient	p-value^†^	Coefficient	p-value^†^
**Years of practice**				
< 5.25	Ref	-	Ref	-
≥ 5.25 to < 12	-1.77	N.S.	-0.91	N.S.
≥ 12 to < 22	7.90	< 0.04	8.34	< 0.03
≥ 22	8.41	< 0.03	9.30	< 0.02
**Percentage of patients on Medi-Cal**				
< 25%	Ref	-	Ref	-
25–50%	-2.41	N.S.	-3.02	N.S.
51–75%	-7.88	N.S.	-8.78	< 0.04
> 75%	5.64	N.S.	4.99	N.S.
**Method of clinician reimbursement**				
Fee for service	Ref	-	Ref	-
Global maternity fee	0.97	N.S.	-	-
Fixed salary	-3.51	N.S.	-	-
**Financial barriers to care**				
Infrequent	Ref	-	Ref	-
Sometimes	-0.18	N.S.	0.79	N.S.
Frequent	7.02	N.S.	8.52	< 0.02
**Duration of visit (min)**				
< 20	Ref	-	Ref	-
≥ 20 to < 30	3.05	N.S.	3.55	N.S.
≥ 30	6.69	N.S.	7.59	< 0.04
**Extent of collaboration with other providers**				
≤ 2	Ref	-	Ref	-
3	4.35	N.S.	4.39	N.S.
4	0.54	N.S.	1.48	N.S.
≥ 5	10.18	< 0.009	10.31	< 0.008
**Adjusted R** ^**2**^	0.209		0.209	

*Adjusted for all variables shown in table

**Adjusted for all variables shown in table except method of clinician reimbursement

^†^N.S. indicates a non-significant result (p-value > 0.05) from overall F-tests

**Table 4:** Characteristics associated with adherence to medical and behavioral/social components: linear regression coefficients and p-values

Sample characteristics	Medical components				Behavioral/socialneeds components			
	Model 1 *		Model 2 **		Model 1 *		Model 2 **	
	Coeff.^‡^	*p*-value^†^	Coeff. ^‡^	*p*-value^†^	Coeff. ^‡^	*p*-value^†^	Coeff. ^‡^	*p*-value^†^
**Years of practice**								
< 5.25	Ref	-	Ref	-	Ref	-	Ref	-
≥ 5.25 to < 12	-0.58	N.S.	-0.19	N.S.	-2.62	N.S.	-1.41	N.S.
≥ 12 to < 22	4.83	N.S.	4.90	N.S.	10.15	< 0.04	10.87	< 0.03
≥ 22	8.51	< 0.04	9.00	< 0.03	8.34	N.S.	9.53	< 0.05
**Percentage of patients on Medi-Cal**								
< 25%	Ref	-	Ref	-	Ref	-	Ref	-
25–50%	0.85	N.S.	0.14	N.S.	-4.81	N.S.	-5.34	N.S.
51–75%	-7.74	N.S.	-9.25	< 0.04	-8.01	N.S.	-8.46	N.S.
> 75%	2.63	N.S.	1.34	N.S.	7.82	N.S.	7.65	N.S.
**Method of clinician reimbursement**								
Fee for service	Ref	-	Ref	-	Ref	-	Ref	-
Global maternity fee	5.39	N.S.	-	-	-2.31	N.S.	-	-
Fixed salary	1.58	N.S.	-	-	-7.23	N.S.	-	-
**Financial barriers to care**								
Infrequent	Ref	-	Ref	-	Ref	-	Ref	-
Sometimes	4.94	N.S.	5.65	N.S.	-3.92	N.S.	-2.76	N.S.
Frequent	6.52	N.S.	7.48	< 0.05	7.42	N.S.	9.29	< 0.05
**Duration of visit (min)**								
< 20	Ref	-	Ref	-	Ref	-	Ref	-
≥ 20 to < 30	3.93	N.S.	4.36	N.S.	2.40	N.S.	2.94	N.S.
≥ 30	0.20	N.S.	0.97	N.S.	11.46	< 0.02	12.45	< 0.008
**Extent of collaboration with other providers**								
≤ 2	Ref	-	Ref	-	Ref	-	Ref	-
3	4.60	N.S.	4.74	N.S.	4.18	N.S.	4.16	N.S.
4	0.11	N.S.	1.31	N.S.	0.86	N.S.	1.60	N.S.
≥ 5	5.25	N.S.	5.23	N.S.	13.78	< 0.007	14.03	< 0.006
**Adjusted R** ^**2**^	0.0497		0.0485		0.23		0.229	

*Adjusted for all variables shown in table

**Adjusted for all variables shown in table except method of clinician reimbursement

^†^N.S. indicates a non-significant result (p-value > 0.05) from overall F-tests

^‡^Regression coefficient

## Results

Figure 2 shows a symmetric distribution of the proportion of components always checked with a mean of 61.7% of components always adhered to, a median of 61.5% and a range of 3.8–100%.

In bivariate analyses, the variables associated with adherence to the guidelines were years of practice (*p* < 0.01), the percentage of patients on Medi-Cal insurance served (*p* < 0.05), the provider method of reimbursement (*p* < 0.05), perceived financial barriers to care (*p* < 0.05), duration of visit (*p* < 0.05) and extent of collaboration with other providers (*p* < 0.01) (Table 2). 

When these covariates were entered into a linear regression model, the only variables that remained significant when adjusting for the other covariates were years of practice and extent of collaboration with other providers (Table 3, model 1). Specifically, clinicians who had between 12 and < 22 years of practice and those with at least 22 years of practice reported on average about 8% more components checked than those clinicians with less than 5.25 years of practice (coefficient *=* 7.90%; *p* < 0.04 and coefficient *=* 8.41%; *p* < 0.03 respectively). Furthermore, those clinicians that collaborated with at least 5 other providers (such as doulas, nutritionists, social workers or psychotherapists) versus clinicians who collaborated with less than 3 providers, also showed significantly higher adherence (coefficient = 10.18%; *p* < 0.009). When we dropped the method of provider reimbursement from the regression model, *additional* variables became significantly associated with overall adherence, namely clinicians whose practices served 51–75% of patients covered by Medi-Cal reported an average of 8.8% fewer components checked than those clinicians who worked in practices that served < 25% of patients covered by Medi-Cal (coefficient*=* -8.78%; *p* < 0.04). In contrast, clinicians who frequently (vs. infrequently) perceived financial barriers to care reported on average 8.5% more components checked (coefficient = 8.52%; *p* < 0.02). Similarly, clinicians who spent at least 30 min with their patients at the first postpartum visit reported on average 7.6% more components checked compared to those who spent < 20 min (coefficient = 7.59%; *p* < 0.04) (Table 3, model 2). For both these models, the adjusted R^2^ remained the same at 20.9%.

Adherence to the medical care components (Mean (SD) = 75.1% (17.2%); Median = 72.7%) was higher than for behavioral and social components (Mean (SD) = 51.9% (23.4%); Median 53.3%) (Supplementary Fig. 1a & 1b).

Table 4 displays the variables that were associated with adherence to medical components and with behavioral/social components in the linear regression models. The full model (model 1) shows that years of practice was the only factor associated with adherence to medical components; specifically, those clinicians with at least 22 years of practice reported an average of 8.5% more components checked than those with < 5.25 years of practice (coefficient = 8.51%; *p* < 0.04). *Additional* factors emerged when dropping method of provider reimbursement from the model (model 2): clinicians whose practices served 51–75% of Medi-Cal insured patients reported significantly less adherence than clinicians whose practices served < 25% of Medi-Cal insured patients (coefficient= -9.25%; *p* < 0.04) while clinicians who perceived frequent financial barriers versus infrequent barriers to care reported on average having checked 7.5% more components (coefficient = 7.48%; *p* < 0.05). The R^2^ for these models at 0.05 was low, indicating that although we found factors associated with adherence, they do not account for most of the variation in adherence. 

Adherence to behavioral/social components was associated with years of practice, duration of the visit and extent of collaboration with other providers; specifically, clinicians with 12 to < 22 years of practice versus those with < 5.25 years of practice reported on average 10% more components completed (coefficient *=* 10.15%; *p* < 0.04) as did clinicians who spent > 30 min with their patients compared with those who spent < 20 min (coefficient = 11.46%; *p* < 0.02) and clinicians who collaborated with ≥ 5 providers versus < 3 providers reported on average almost 14% more components completed (coefficient = 13.78%; *p* < 0.007) (Table 4, model 1). *Additional* factors surfaced when dropping method of provider reimbursement (Table 4, model 2): at least 22 years of practice also showed higher adherence compared to < 5.25 years of practice (coefficient = 9.53%; *p* < 0.05) as did perceived frequent financial barriers to care (coefficient = 9.29%; *p* < 0.05) (Table 4, model 2). The R^2^ for these models was 0.23.

Notably, years in practice were not directly associated with any external barrier. However, it did modify the association between the number of collaborators and adherence. Among clinicians with 12 or more years of practice, having collaborations with 5 or more providers allowed for higher adherence. Whereas among clinicians with < 12 years of practice, collaboration with many providers did not significantly increase adherence (*p* < 0.001).

## Discussion

One of the goals of professional practice guidelines is to assist clinicians in performing evidence-based, comprehensive, continuous care. Comprehensive postpartum care, following the ACOG bundle, can reduce maternal mortality and morbidity, the majority of which takes place in the vulnerable postpartum period [4, 18]. It follows that the more comprehensive adherence is to the guidelines, the more effective the care team will be at reducing morbidity and mortality. According to our findings, clinicians reported that at the first postpartum visit, on average, they always checked 62% of the listed care components recommended by ACOG. Adherence was lower for behavioral/social components than medical components. A previous study of obstetrician/gynecologists in Louisiana found large variations in the implementation of specific ACOG recommendations ranging from 46.8–98.4%. [7] Adherence to ACOG guidelines at the first *prenatal* visit is higher according to a 2019 study which reported a median score of 74% [19]. Full adherence to postpartum guidelines is possible given that a handful of clinicians in our study reported 100% adherence. Nonetheless, as more behavioral care and social aspects of care recommendations are incorporated to address whole-person care in the postpartum, follow-up on these recommendations becomes more challenging for clinicians [6]. 

We found that a combination of internal and external factors was associated with adherence. More years in practice, an internal factor, was an important predictor of overall adherence, even after controlling for covariates; clinicians with at least 12 years of practice reported significantly more adherence than those with approximately 5 years of experience or less. Added years of experience likely translates into more knowledge, skills, efficiency and ease of collaboration with multiple providers, assets that facilitate the implementation of guidelines. It may also be an indicator of more holistic care practices and better disposition to confront challenges.

Four external factors were associated with overall adherence: collaboration with five or more providers -- an indicator of a robust integrated practice or greater connection to community resources– and a longer visit duration, markedly increased adherence at the first postpartum visit when controlling for covariates. The perception of high financial barriers to care also increased adherence. Underinsurance or breaks in coverage may incentivize clinicians to provide more holistic care during the first postpartum visit, since they may be aware of patients’ poor access to timely follow-up care. A longer visit suggests that with more time available to clinicians, they can check more components. However, the reverse may also be true: increased patient needs may require more clinician time. We are unable to ascertain what is going on with our cross-sectional data. In contrast, clinicians who saw a rather high concentration of patients covered by Medi-Cal (51–75% vs. ≤25%) reported lower adherence, when controlling for other covariates. Mixed practices that serve members of both commercial and Medi-Cal health plans often must manage different reimbursement schemes with different care requirements. Under these conditions, clinicians must make tradeoffs, and some care components may fall by the wayside. Notably, clinicians whose practice settings served 75–100% of Medi-Cal insured patients did not show significantly different levels of adherence compared with clinicians serving < 25% of Medi-Cal insured patients. This could be because clinicians responding that they served very large volumes of Medi-Cal insured patients were practicing predominantly in community hospitals/clinics or federally qualified health centers which might be better equipped to care for disadvantaged populations compared with academic settings, private practice or staff model HMOs. This is an issue requiring further research. Medi-Cal, which pays for 39% births in California is being transformed [20]. It has extended coverage from 60 days postpartum to 12 months postpartum, eliminating premiums for families, expanding doula and community health worker-based services and, demonstrating commitment to supporting whole-person care [20]. 

Upon examining adherence to specific groups of care components, we found that longer years of practice and perception of frequent financial barriers increased adherence for both medical and behavioral/social components. In contrast, a mixed practice of 50–75% Medi-Cal insured patients and 25–50% commercially insured patients, reduced adherence only for components of medical needs, whereas the extent of collaboration with other providers and visit duration were associated only with adherence for behavioral/social components. The latter findings suggest that more time and collaboration with other providers is required to complete behavioral/social components. Future studies need to explore whether competing values or other unexamined factors such as discomfort with addressing social-behavioral issues, or regulations that prioritize the assessment and screening of medical versus social-behavioral components diminish whole-person care.

### Strengths and limitations

Our study had several strengths, including a high response rate and diversity in racial, age and practice characteristics of the study clinicians. We assessed their overall adherence to ACOG guidelines and to specific broad categories of care components, instead of adherence to single clinical components, to understand the extent to which comprehensive whole-person care is being provided in the postpartum. Guided by a conceptual framework, we were able to identify some important factors associated with adherence to the guidelines as indicated by the reasonable robustness of our models. Our study was not designed to assess faults in the guidelines or familiarity with the guidelines, the psychological incentives to comply with guidelines, or their application under different clinical circumstances.

The study had some limitations. Our voluntary sample does not allow us to generalize to all practicing clinicians in California [6]. Our sample included more OB/GYNs that were females, younger than 35 years of age and white non-Hispanics compared with the active OB/GYN population in California [14]. Furthermore, our sample included similar proportions of white non-Hispanic midwives as those licensed in California, but fewer Black and Hispanic midwives. (15–16) The small sample size did not allow us to examine a larger number of potential factors associated with adherence. Further, this outcome was based on self-report rather than direct observation or clinical records. Finally, we only focused on the degree of adherence achieved in the first postpartum visit and did not capture the care provided on subsequent visits.

### Clinical implications

Women in the postpartum period are experiencing rising morbidity and mortality [18]. Evidence-based clinical practice guidelines help address these problems, but only effectively if they are adhered to as a comprehensive bundle, which roughly half the time they are not. We sought to understand what factors are associated with guideline adherence.

Perhaps contrary to expectations, our study did not find that less experienced, fresh from training clinicians, assess and screen patients better than more experienced clinicians. A study of hospitalists caring for Medicare patients showed slightly better outcomes when the physicians were younger, and the authors suggested more continuing medical education was needed, presumably to help older clinicians follow the latest guidelines [21]. We found the opposite, that more years of practice may allow the clinician to do more and be more efficient, collaborate better with others through evolved networks; and that these skills were directly related to guideline adherence. Ease of collaboration is backed up by our data: whereas among clinicians with 12 or more years of experience, having collaboration with 5 or more providers allowed for high adherence, this was not the case for less experienced clinicians. Our medical training system assumes that right after completing training a physician is at their most knowledgeable and continuing medical education is needed to mitigate the loss of this knowledge. In this article, we are able to demonstrate the value of experience and how it improves the quality of care through guideline adherence.

Historically more than half of Medi-Cal recipients have trouble finding a physician who will offer them care, and that percentage is even higher in Medi-Cal managed care plans. (22–23) This can set up a vicious cycle of patients with deferred care needs not being able to access care, which creates a challenge in providing comprehensive care for these patients once they are accepted into care. We found that clinicians who serve an overwhelming majority of Medi-Cal insured patients reported similar levels of adherence to those serving very few Medi-Cal insured patients. So, while Medi-Cal insured patients might have greater social-behavioral needs, focusing on this population allowed clinicians to overcome that barrier and provide comprehensive care.

Postpartum care traditionally has been limited to one visit offered 1–2 months after birth. ACOG guidelines and other studies show that this is insufficient to lower morbidity/mortality and inequities in this vulnerable period. A growing understanding of the needs after a delivery has been supported by public policy and the extension of Medi-Cal and other state’s benefits for a full year. But these benefits may not improve outcomes unless evidence-based guidelines are followed. Clinicians will struggle to meet these guidelines if they themselves are inexperienced, have overly complicated practices or reimbursement schemes, or multiple patient populations. Previously we found that clinicians facing barriers will make trade-offs in care without the benefit of tools or priorities to guide those trade-offs [6]. Patients will therefore likely receive less comprehensive care.

Some suggestions to improve adherence include making guidelines more accessible at the point of care through EMR or other computerized clinical decision supports systems that link guidelines to reimbursement (i.e. CPT codes); and having clinicians partner with more experienced providers. Continuing medical education requirements should focus more on those skills that come with experience which further study might elucidate. In addition, practices that are structured to collaborate with multi-disciplinary providers such as social workers or doulas would likely do better at meeting the entirety of guideline recommendations for the most comprehensive care.

## Conclusion

Clinical practice guidelines are an essential tool to decrease morbidity, mortality and inequities in the vulnerable postpartum period. When ACOG revised their postpartum care guidelines in 2018, the goal was to provide ongoing, individualized care that encompasses medical, behavioral and social needs and to use a bundle approach that had been shown to be effective in reducing maternal morbidity and mortality. If clinicians were to comprehensively adhere to the guidelines, they could reverse the rising tide of maternal morbidity and mortality. We sought to understand which clinicians were more able to comprehensively meet the guidelines, starting at the first postpartum visit, and found that more experienced clinicians, those that simplified their practices to either all Medi-Cal or few Medi-Cal recipients, and those that collaborated more with other providers were best able to provide the care recommended in the guidelines. Our findings highlight where policy, resources and training are needed to improve guideline adherence and whole person care.

## Electronic supplementary material

Below is the link to the electronic supplementary material.

Supplementary Material 1

Supplementary Material 2

## Data Availability

The data analyzed during the current study are mostly included in this article and its supplementary files. Data are available from the corresponding author upon reasonable request.

## Abbreviations

ACOG: American College of Obstetricians and Gynecologists
Medi-Cal: Medicaid insurance coverage in California
ACEs: Adverse childhood experiences
OB/GYN: Obstetrician and Gynecologist
HMO: Health Maintenance Organization
N.S.: Non-significant result at an alpha level of 0.05
EMR: Electronic medical record
CPT: Current Procedural Terminology
