# Supplementary figures and images for: Factors associated with clinician adherence to guidelines for postpartum care: results from a California survey
Source: BMC Pregnancy Childbirth. 2025 Mar 13;25:283. doi: 10.1186/s12884-025-07362-0 (PMC11907915; doi:10.1186/s12884-025-07362-0)

**Supplementary Figure 1a.** Proportion of medical care components that clinicians always check


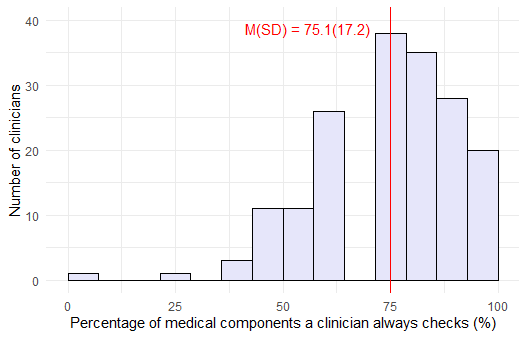

Supplement: Supplementary file 1 — Supplementary Material 1 [file 12884_2025_7362_MOESM1_ESM.docx]
